# An Ecological Paradox: The African Wild Dog (Lycaon Pictus) Is Not Attracted to Water Points When Water Is Scarce in Hwange National Park, Zimbabwe

**DOI:** 10.1371/journal.pone.0146263

**Published:** 2016-01-27

**Authors:** Henry Ndaimani, Paradzayi Tagwireyi, Lovelater Sebele, Hillary Madzikanda

**Affiliations:** 1 Department of Geography and Environmental Science, Geo-information and Earth Observation Centre, University of Zimbabwe, Mount Pleasant, Harare, Zimbabwe; 2 Zimbabwe Parks and Wildlife Management Authority, Causeway, Harare, Zimbabwe; 3 Painted Dog Conservation, Hwange National Park, Dete, Zimbabwe; University of Southern Queensland, AUSTRALIA

## Abstract

In dry biomes, spatio-temporal variation in surface water resource stocks is pervasive, with unknown effects on the ranging behaviour of large predators. This study assessed the effect of spatial variation in surface water resources on the ranging behaviour of the African wild dog (*Lycaon pictus*). We analyzed data for 1992 (dry year with 20 water points) and 2000 (wet year with 30 water points) against presence-only data for five packs of *L*. *pictus* in a part of Hwange National Park and adjacent smallholder communal farming areas in western Zimbabwe. Modelling the potential habitat for *L*. *pictus* using Maxent with distance from water points (D_w_) and Normalized Difference Vegetation Index (NDVI) as predictor variables was successful for 2000 (AUC = 0.793) but not successful for 1992 (AUC = 0.423), with *L*. *pictus* probability of occurrence near water points being more for year 2000 than for year 1992. The predicted *L*. *pictus* range was wider in 1992 (~13888.1 km^2^) than in 2000 (~958.4 km^2^) (Test of Proportions, χ^2^ = 124.52, df = 1, *P* = 0.00). Using the 2^nd^ order Multitype Nearest Neighbour Distance Function (Gcross), we also observed significant attraction between *L*. *pictus* and water points within only ~1km radius for 1992 but up to ~8km radius for 2000. Our study reinforced the notion that surface water resources attract wild dogs in the savannahs but paradoxically less so when water resources are scarce. In particular, our study furthers current understanding of the effects of changing water availability regimes on the endangered *L*. *pictus*, providing evidence that the endangered predator’s home range encroaches into potential ecological traps (i.e., smallholder communal farming areas) when water resources are scarce.

## Introduction

In dry biomes, the relationship between wildlife (particularly large mammals) and surface water resources is well documented (e.g., [1, 2–6]). In tandem with the resource gradient hypothesis (sensu [7]) reviewed in [8, 9] water is a key driver of biological diversity and population dynamics [10–12]. In particular, water stress is regarded as a primary factor regulating the distribution and abundance of fauna in dry biomes (e.g., bighorn sheep (*Ovis candadensis*), camel (*Camelus spp*), dingoes (*Canis lupus dingo*) [13–15] and ungulates migration in the Serengeti [16, 17]). Wildlife (e.g., large ungulates) were observed to be more attracted to water when water resources were scarce than when water resources were abundant [18, 19]. Consequently, wildlife managers have (since the 1940s) developed surface water resources in dry environments to enhance wildlife habitats [20, 21].

The availability of surface water is a major driver of selected ungulate distribution in most dry wildlife areas [22–24]. This is particularly true for grazing animal species [e.g., impala (*Aepyceros melampus*)] that need to drink water on a daily basis [25, 26] and carnivorous animal species (e.g., dingoes) that do not need to drink water daily [27]. However, water is not an important factor driving the distribution of some dry-biome animals e.g., bighorn sheep [13] and oryxes (*Oryx leucoryx*) [28, 29]. While surface water may directly influence the distribution of ungulates [19, 30], it is likely to indirectly influence the distribution of key predator species of those ungulates. For example de Boer [3] reported that the spatial distribution of lion (*Panthera leo*) kills was determined by the water dependency of prey species in Klaserie Private Nature Reserve, South Africa. Also, Arjo *et al*. [6] speculated that additional surface water sources relaxed the arid limitation for coyotes (*Canis latrans*) in the Great Desert Basin, Utah, USA.

In the African savannahs, a wet year with total annual precipitation that is above long term average, provides ample surface water resources for wild animals [31]. Additionally, in wetter years, the amount of preformed water available to both prey and predators populations likely increases [32]. However, during a dry year, most water points dry up so there is increased competition for water and other resources at the few remaining viable water sources [19, 26]. As such, surface water is a key driver of wildlife distribution during dry years as grazing centres around water points as a copying strategy to enhance access to the water [30, 33]. Consequently, predators [e.g., lion, cheetah (*Acinonyx jubatus*), spotted hyena (*Crocuta crocuta*), African wild dog (*Lycaon pictus*) etc.] may be attracted to these water points as a strategy to enhance hunting success [30, 33, 34]. However Groom and Harris [35] found that the availability of surface water had no significant effect on the likelihood of grazers being present, even in the dry season, suggesting that the relationship between wildlife and water resources may not necessarily be straightforward for all fauna.

In addition to water, numerous other factors have been reported as important drivers of wildlife distribution in the savannah ecosystem. For example: Matawa *et al* [36] reported that the distributions of the African elephant (*Loxodonta africana*) and the buffalo (*Syncerus caffer*) are driven by human landscapes (e.g., roads and human settlements) in the Sebungwe region, Zimbabwe. Also, Winterbach *et al* [37] reported that the distribution of the large carnivore guild consisting of lion, leopard (*Panthera pardus*), brown hyena (*Hyaena brunnea*), and cheetah depends primarily on prey availability, interspecific competition, and conflict with humans in Botswana. Additionally, Ogutu *et al* [38] reports the availability of water and human settlements as major drivers of big herbivore distribution in the Mara region of southwestern Kenya. Understanding the factors that drive wild fauna distribution is an important step in wildlife conservation particularly endangered species in the savannahs, [39, 40]. This study investigates how the availability of water resources influence the distribution of the African wild dog in the savannahs.

The African wild dog (*hereafter* wild dog) is a medium sized predator [41] whose population has largely been extirpated in most of Africa’s wilderness [42, 43]. Recent studies suggest that the wild dog populations in protected areas continue to fall despite increasing efforts to conserve the predator [44]. Consequently, many studies have attempted to understand the predator’s feeding, breeding and roaming behaviour, conflict with humans, and kleptoparasitism in an effort to enhance its conservation status (e.g., [45, 46–51]). While studies that test the distribution of the wild dog are many (e.g., [52, 53, 54]), few of these to the best of our knowledge, explain how the distribution and viability of surface water resources could contribute to wild dog conservation.

In this study, we tested whether or not the occurrence of wild dogs is explained by the distribution of surface water resources in the Hwange National Park and adjacent smallholder communal farming areas, north western Zimbabwe. We particularly asked whether attraction intensity of wild dogs to surface water points was the same for two dry seasons with noticeable differences in number of viable water points. We hypothesized that wild dog attraction to water points would increase when there are less water points. To test this hypothesis, we used data from years exhibiting contrasting surface water availability conditions (i.e., 1992 and 2000).

## Materials and Methods

### Ethics statement

Handling of wild dogs for GPS collaring and data collection for this research was approved under DM 173: PERMIT # 11(1)(C)(2)2000 issued by the Zimbabwe Parks and Wildlife Management Authority.

### Study site

**Fig 1:**
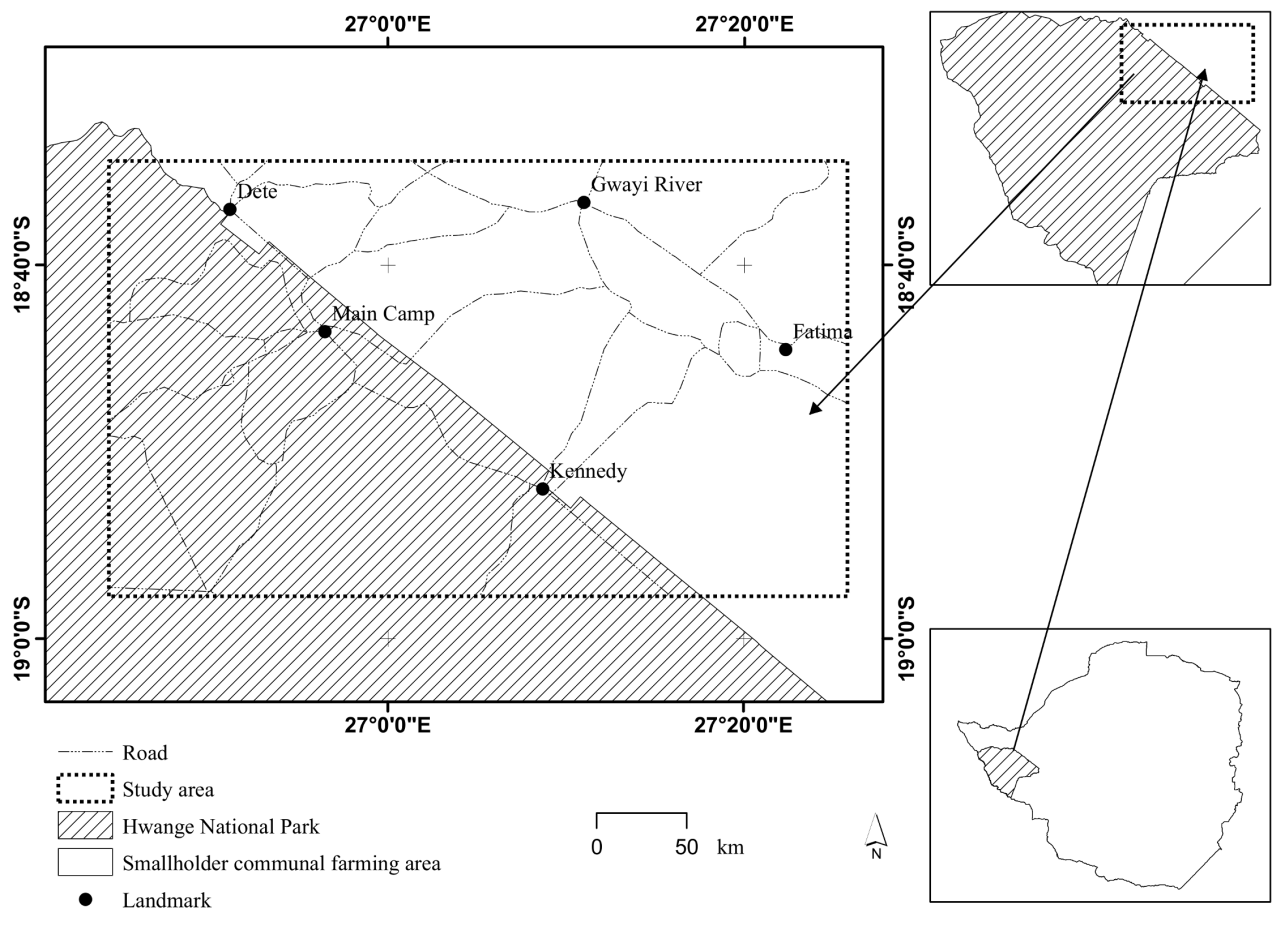
Location of the study system comprising of part of the Hwange National Park and adjacent communal smallholder farming areas in Zimbabwe

Our study system was a 3128km^2^ rectangle comprising partly of the Hwange National Park and adjacent smallholder communal farming areas (*hereafter* communal farming areas) in north western Zimbabwe. The study system lies between latitudes 18.57°S– 18.96°S and longitudes 26.73° E– 27.43°E (Fig 1). Most of the study site lies on Kalahari sandy veld with vegetation dominated by African teak (*Baikiaea plurijuga*), Silver cluster-leaf (*Terminalia sericea*) and bushwillows (*Combretum* spp) [55]. Common herbivore species in the study system include: elephant, buffalo, impala, giraffe (*Giraffa camelopardalis*), sable (*Hippotragus niger*), and kudu (*Tragelaphus strepsiceros*) [56]. Large predators in the area include: lion, spotted and brown hyena and leopard. The ephemeral Gwayi River system is the major drainage feature cutting through the study area, with riparian vegetation covering the mainly alluvial soils dominated river valley. The system is prone to drought, with precipitation largely in the form of rainfall (long-term mean of 606mm), received between October and April [57]. Hence the area experiences dry and wet years making it interesting to monitor the wild dog ranging behaviour in response to contrasting conditions of water stress. The mean annual temperature for 1992 ($\bar{⨯}$ = 23°C, σ = 3.87) and year 2000 ($\bar{⨯}$ = 21.42°C, σ = 3.47) were almost the same, so we expected the ranging behaviour of wild dogs not to be influenced differently by temperature for the two years periods.

The area generally lacks natural surface water due to its climate and geology. During the dry season (May to September) artificial water points (pumps that draw water from the aquifer) are maintained to provide drinking water to fauna [58]. During years when rainfall exceeds the long term average, it is expected for all water points to be viable but during a drought year some water points dry up. As such, the study system provides ideal conditions to test our hypothesis. For example it is possible to know the number and location of viable water points during any dry season. We also considered this area as ideal for this study because it lies within the known range of five packs of Global Position System (GPS) collared wild dogs. The wild dogs in our study system breed (have pups) in May–July in ground burrows (dens) [59]. Before the pups are weaned, wild dogs, which are group breeders, abandon their nomadic way of life and become sedentary canids that hunt within the locality of their dens to nourish the pups [46]. By the end of August the pups at about eight weeks old are weaned and the packs resume their nomadic way of life [59].

### MaxEnt modelling

Firstly, we obtained the month of September presence-only location data of wild dogs from five GPS-collared wild dog packs with home ranges that overlap with the study area (S2 Table). The wild dog packs were fitted with GPS collars during the period from 1991 to 2002 by Painted Dog Conservation as described by van der Meer *et al*. [57]. At least one pack member from all packs occurring in the study area was GPS-collared during both periods. Our analyses are restricted to the dry season fixes of wild dogs for the month of September recorded in 1992 and 2000 because we wanted to document the ranging behaviour of wild dogs during a time when surface water availability was limited to artificial water points and the wild dog packs will have returned to their nomadic way of life after weaning pups. The GPS fixes followed a random pattern for both day and night to cover the wholesome ranging behaviour of the wild dogs.

Secondly, we computed normalised difference vegetation index (NDVI) [60] from Landsat TM and Landsat ETM images for the month of September 1992 and 2000 for the study area. We used the images of the month of September (dry season) because the wild dog location data used in our analyses were collected during the dry season. NDVI is a proxy for vegetation vigour where high values represent green vegetation whereas low values represent dry vegetation [61]. We used vegetation vigour in our analyses because we assumed that herbivores and specifically wild dog prey species select areas of high forage quality and quantity. The foraging behaviour of wild dogs was assessed during the dry season at a time when most vegetation is dry and leafless and herbivores are likely to be attracted to the green vegetation patches.

Thirdly, we calculated the distance of locations from the nearest water point (*D*_*w*_) using the Euclidian distance algorithm in ArcGIS (Environmental Systems Research Institute: Redlands, California, USA). We obtained the study area data (shapefiles) of the distribution of water points from the monthly monitoring database made available by the Zimbabwe Parks and Wildlife Management Authority (ZPWMA). In most of the communal farming areas where surface water data were not readily available, water points were digitized from high resolution satellite images freely available on the Google Earth platform. Twenty viable water points in year 1992 (drier year, mean annual rainfall = 579.4mm) and 30 viable water points for year 2000 (wet year, mean annual rainfall = 826.3 mm) were used in our analyses (S1 Table).

Fourthly, we built two models (the first for 1992 and the second for 2002) predicting the potential distribution of wild dogs based on NDVI and distance from water using Maximum Entropy (MaxEnt) [62, 63]. The MaxEnt algorithm models the potential distribution of target species based on presence-only data and a set of relevant environmental variables. In this study, wild dog location data collected in the study area in 1992 and 2000 were used as the presence only data while distance from surface water and NDVI data described in previous sections were used as the explanatory variables. We selected MaxEnt for our analyses because it has been shown to perform better than other candidate spatial distribution models [64].

### MaxEnt model evaluation

We evaluated the predictive ability of each of the two MaxEnt models using the Area Under Curve (AUC) of the Receiver Operating Characteristic (ROC) curve technique [65, 66]. The response of wild dogs to distance from surface water and NDVI was tested using the response curves for both MaxEnt models. In addition, the individual contribution of distance from water and NDVI to the model was obtained from the analyses of variable contribution results in MaxEnt. We also used the logistic threshold of equal training and test sensitivity to produce a binary map showing potential presence and absence of wild dog for both 1992 and 2000. We then determined areal extent of the predicted distribution using the area calculation algorithm in ArcGIS.

### Statistical analyses

**Fig 2:**
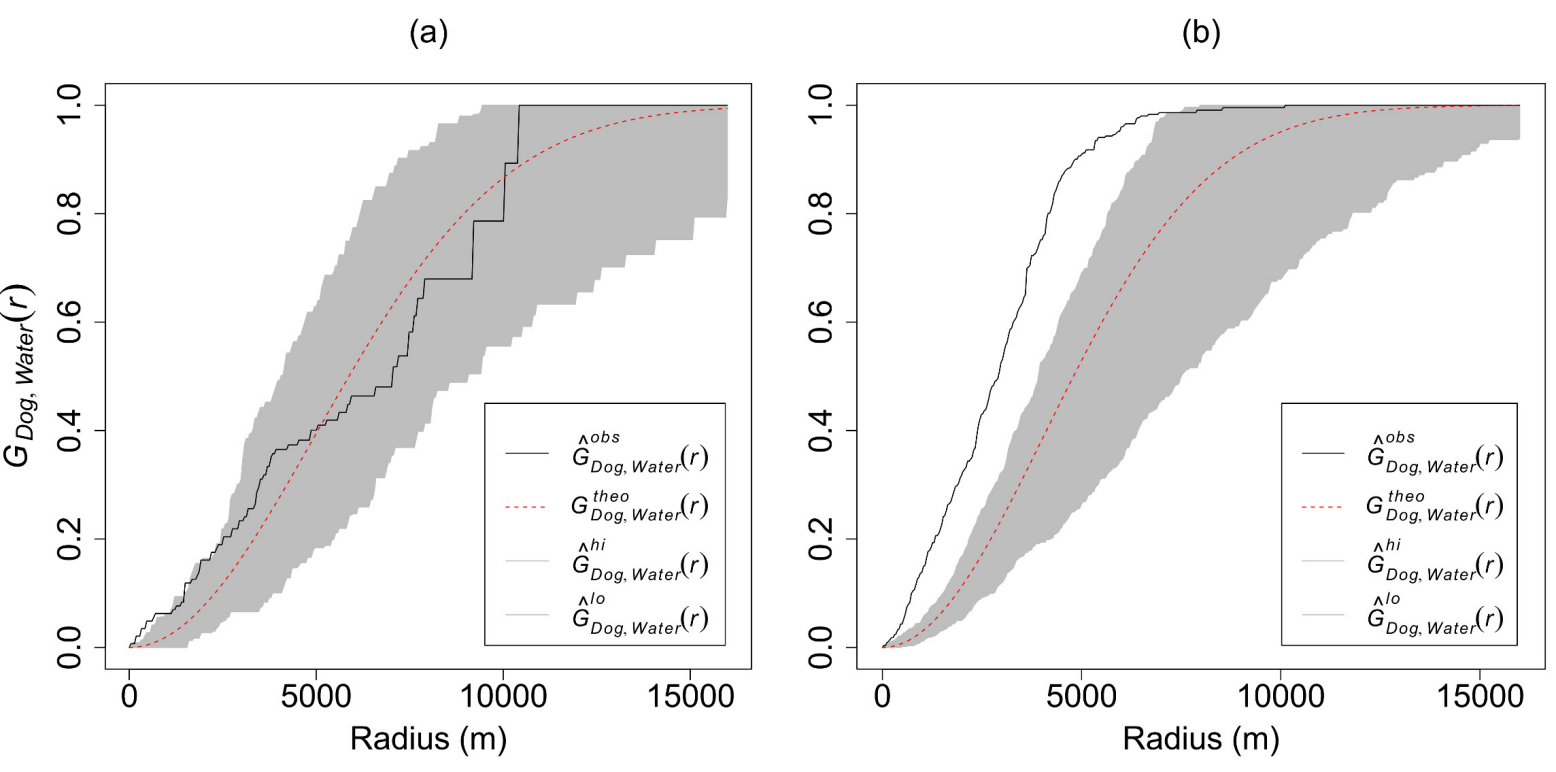
2^nd^ order Multitype Nearest Neighbour Distance Function (Gcross) graphs showing significant spatial aggregation of wild dogs (a) within ~1km for 1992 (drought year), and (b) within ~8km for 2000 (wet year) radii around water points (observed pattern ≥ the simulation 95% confidence interval envelopes). ${\hat{G}}_{Dog,water\;(r)}^{obs}$ = observed Gcross function; $G_{Dog,water\;(r)}^{theo}$ = theoretical Gcross function; shaded grey area = 95% confidence interval around the theoretical Gcross function and; ${\hat{G}}_{Dog,water\;(r)}^{hi/lo}$. = upper and lower confidence level about the theoretical Gcross function.

We used the 2^nd^ order Multitype Nearest Neighbour Distance Function (Gcross) [67] implemented in R software [68] to analyze spatial aggregation of wild dogs at water points using the wild dog and water points data for 1992 and 2000. We used the 2^nd^ order Gcross function because we assumed that wild dogs are territorial [69]. To test for significance of spatial aggregation, we generated 95% Confidence Interval (CI) simulation envelopes under Complete Spatial Randomness (CSR) using 499 simulations following Perry et al. [70]. We then deemed significant aggregation of wild dogs to water points as occurring when the observed line (of the Gcross function) was above the simulation envelopes. We also considered significant segregation to occur when the observed line was below the simulation envelopes (Fig 2). To test for difference in the size of the predicted wild dog potential distribution between 1992 and 2000, we used the proportions test (χ^2^ test) described by Wilson [71] which we implemented in R software.

## Results

The test for spatial aggregation of wild dogs at water points using the Gcross function showed significant attraction only within ~1.0km radius for the 1992 data, but to within~8.0km radius for the 2000 data (observed pattern ≥ the simulation 95% confidence interval envelopes; Fig 2).

**Fig 3:**
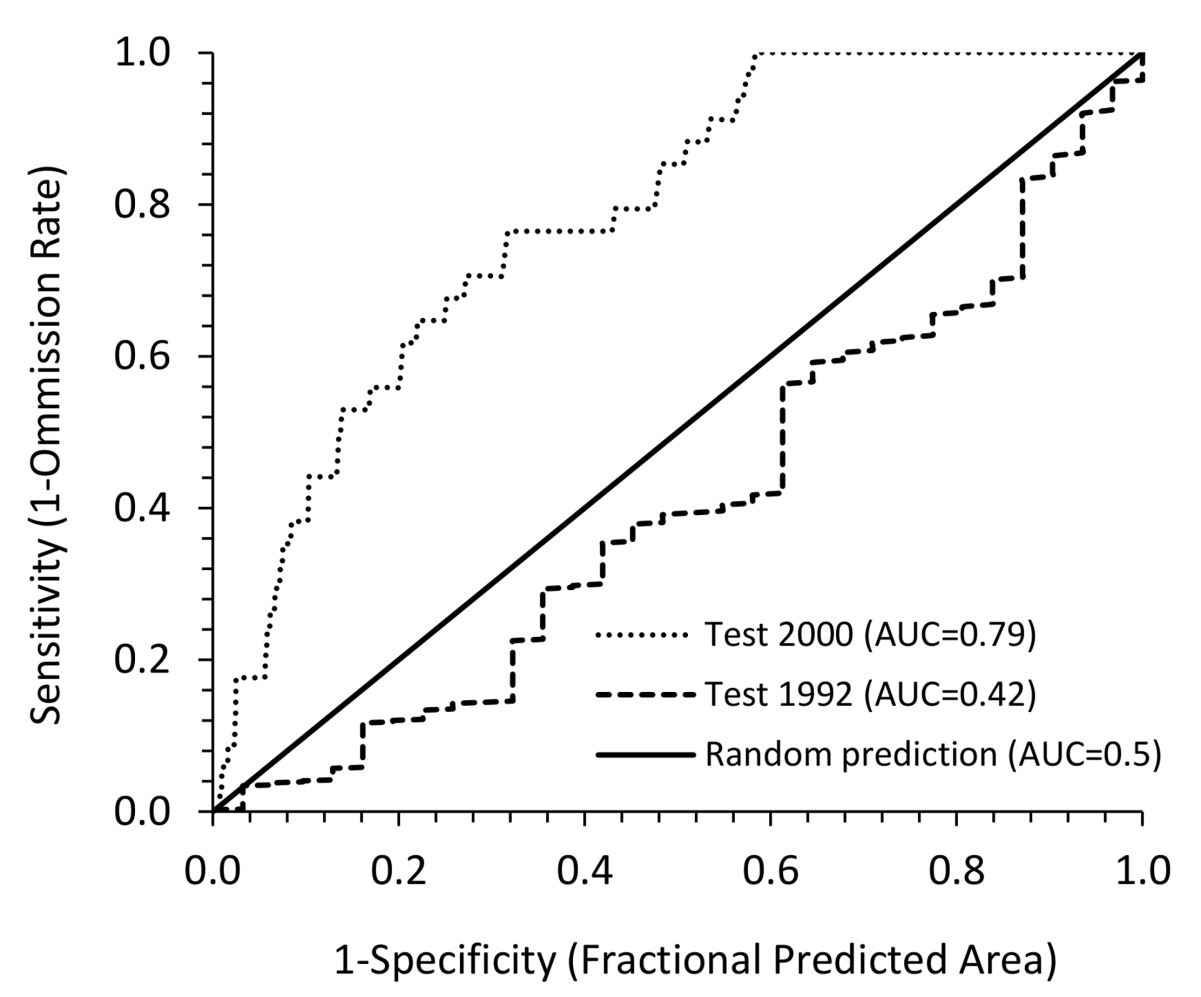
Area Under Curve (AUC) of the Receiver Operating Characteristic (ROC) curves for the MaxEnt habitat models based on wild dog presence only data showing not successful prediction for 1992 (dry year) and successful prediction for 2000 (wet year).

MaxEnt modelling with *D*_*w*_ and NDVI as environmental variables successfully explained the distribution of wild dogs for the year 2000 (AUC = 0.793) but failed to do so for the year 1992 (AUC = 0.423) (Fig 3). The MaxEnt results also showed that the contribution of surface water to the models was 79.3% in year 1992 and 90.6% in year 2000.

**Fig 4:**
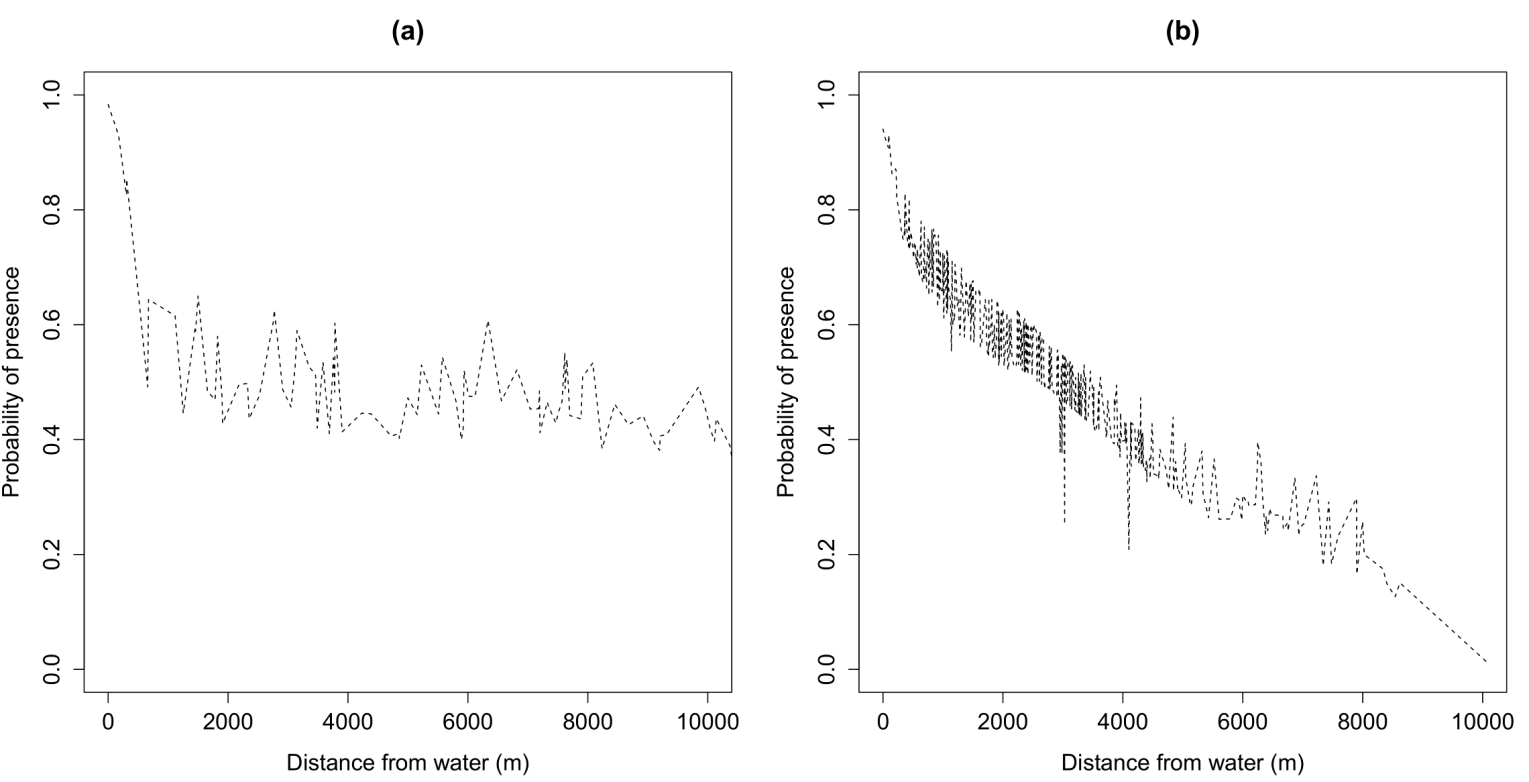
MaxEnt derived response curves showing lesser influence of water on the probability of wild dog presence in (a) 1992 (dry year) than in (b) 2000 (wet year).

MaxEnt results also revealed lesser influence of water points to the probability of occurrence of wild dogs with increasing distance from water for 1992 than for 2000 (Fig 4).

**Fig 5:**
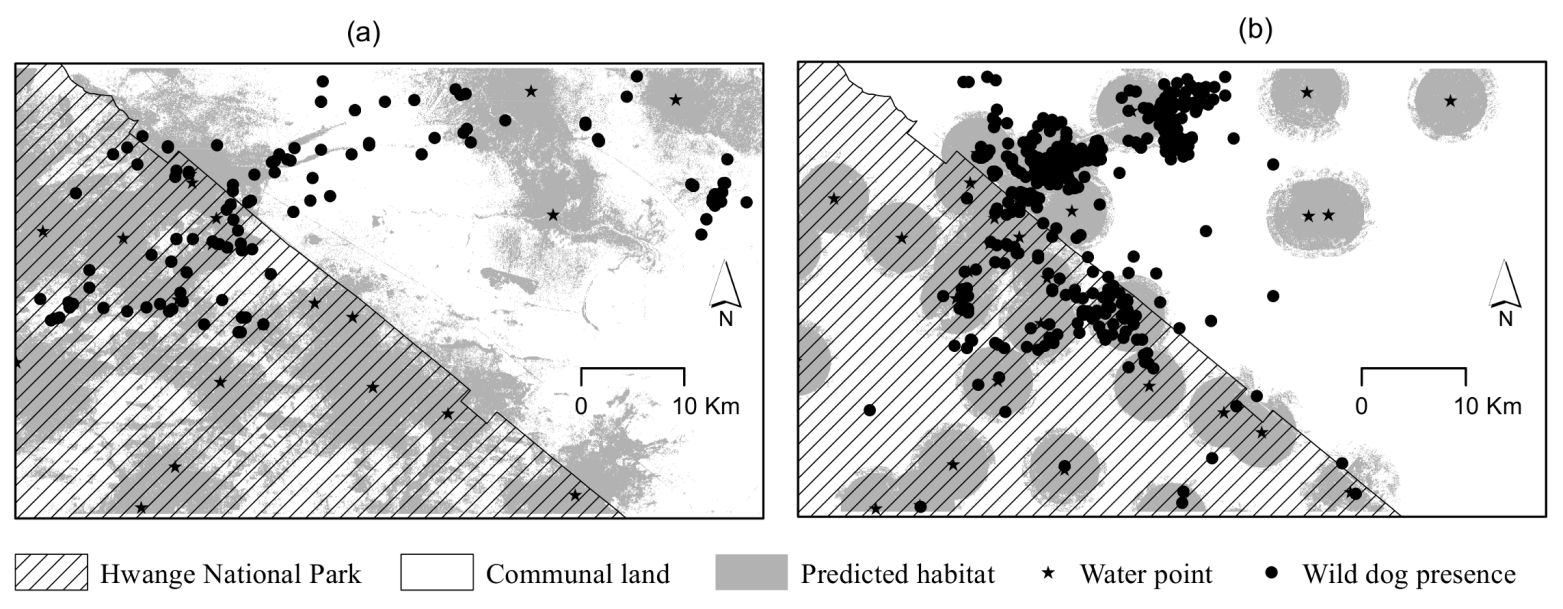
Pictures of MaxEnt models showing wider predicted wild dog habitat in (a) 1992 = dry year (~13888.1 km^2^) than (b) 2000 = wet year (~958.4 km^2^), together with wild dog presence data and water points.

We also observed that the area predicted (using MaxEnt) as wild dog habitat was larger (~13888.1 km^2^) in 1992 than (~958.4 km^2^) in 2000 (Z test: χ^2^ test = 124.52, df = 1, p < 0.0001), representing a drop from 44.3% of the study area to 30.6% (Fig 5).

## Discussion

Contrary to our expectations that wild dogs would aggregate more around water points when water points are few than when water points are more, our findings suggest that wild dogs aggregate more near water points when water resources are abundant than when they are scarce (see Fig 4). These findings support previous research e.g., Arjo *et*. *al* [6] who reported amplified coyote activity around water points when water sites were increased in the Great Basin Desert, Utah. However, our findings contradict Hall *et al* [32] who found that coyote activity was not higher near water sources. Abundance of water points in the landscape explained distribution of wild dogs during the wet year whilst its effect was absent during the dry year. In particular, results from the MaxEnt models suggest higher chances of wild dog occurrence near water points than further away when water resources were abundant than when they were scarce. Distribution maps from the model also suggest that wild dogs generally ranged wider when water resources were scarce than when they were abundant.

The high probability of occurrence of wild dogs near water points during wet years could be explained by the ‘resource gradient hypothesis’, which suggests that the territory of a given animal species depends on the dispersion pattern of its prey [9]. Impalas, which are the main prey species of wild dogs in savannah landscapes [52, 53] are water dependent [72]. Consequently, the distribution of the wild dog could be driven by the occurrence of the impala which in turn is driven by the spatial spread of drinking water sources [73, 74]. Additionally, the wild dogs may also be attracted to the water because they need to drink themselves.

Paradoxically, observations made during the drier year (1992) suggest the opposite of the ‘resource gradient hypothesis’ where wild dogs show less attraction to water points and also range far (Fig 5). Dispersion of wild dogs from their key prey areas could be explained by intraguild competition [e.g., (intraguild predation: the predation of mesopredators by apex predators [75, 76]) and (kleptoparasitism, the competition for kills among predators [50, 77])]. Apex predators (e.g., lions and hyenas) may suppress wild dogs both by killing them, or instilling fear, which motivates changes in behaviour and habitat use that limit mesopredator (e.g., wild dog) distribution [78–81]. It is therefore possible that the key prey areas have high densities of the apex predators during the drier year hence the wild dogs (being weak competitors) are driven away (i.e., predation and kleptoparasitism avoidance). Consequently, the wild dogs retreat to communal farming areas where competition is minimal [82]. Communal farming areas offer an opportunity for easy prey in the form of domesticated animals. If that is the case, then these findings made for the dry year also support the “resource gradient hypothesis” where the ranging behaviour of wild dogs during this time could be explained by distribution of domestic prey. However communal farming areas are potential ecological traps to wild dogs as farmers are likely to persecute the dogs for livestock kills [57].

Our study however did not test for the evidence and magnitude of possible wild dog kleptoparasitism competition with sympatric carnivores in the study area during both the dry and wet years. That analysis was not possible because we did not have data on the density of the large predators that compete with the wild dogs in the study area and data on kills and kill sites as well. Future studies that further seek to explain the effect of water on the ranging behaviour of wild dogs should therefore include analyses on how the densities of lions and hyenas in the study area affect the ranging behaviour added to the effect of water. In addition, our model predicting the distribution of wild dog was based on only two predictors (vegetation cover approximated by NDVI and distance from surface water). Inclusion of other key variables like the density of prey species could improve the predictive ability of models. While herbivore population data is available for the park [83], it is largely not available for the communal areas which also form part of the study site. Despite these shortfalls, we maintain that the two factors used in our models are key in driving the distribution of prey species [84] and could therefore be used as proxies for prey species density. We also appreciate that our focal species is territorial and their ranging behaviour is likely influenced by the desire to minimize encounters with other packs [59], therefore further studies on the ranging behaviour of wild dogs should include the effect of territoriality.

Our study is unique in that it is the first to objectively test the effect of surface water distribution on the ranging behaviour of wild dogs in African savannahs. The study is also amongst the first to employ spatial analyses methods in answering hypotheses on surface water influences on wild dog ranging behaviour. Results from the study have far reaching implications on the conservation of the wild dogs and other canids since availability of surface water has been known to increase kleptoparasitism and thus threaten wild dog populations [52]. For example, possible management interventions would include provision of more water points in the landscape in an effort to reduce intraguild competition. This study could therefore form the basis for formulation of future hypotheses that test the effect of surface water distribution on wild dogs and other canids. Future studies exploring similar hypotheses should increase spatial replication to include several landscapes. This will better enable generalisations to be made with regards to the effect of surface water on the ranging behaviour of wild dogs.

## Conclusions

Our results indicate that availability of surface water influences the habitat preferences of the African wild dog in two opposing ways. Firstly, as expected and in tandem with the ‘resource gradient hypothesis’ there is evidence of attraction between surface water and wild dog distribution during wet years. The mechanism of the attraction could be that wild dog prey (e.g., impala) aggregate around water points and wild dogs follow prey herds for their nutrition. Secondly, in complete disagreement with the ‘resource gradient hypothesis’, there is evidence that wild dogs are not attracted to water points when water points are few. This paradox may be explained by the intraguild predation and kleptoparasitism concept. During prolonged dry period (droughts), some water points dry up, consequently there is more concentration of prey around the remaining water points. This concentration of prey also attracts apex predators (e.g., lion and hyena) which outcompete wild dogs for kills and possibly prey on wild dogs. As such wild dogs are driven away from near the water points to peripheral areas free from competition for kills and danger of predators. We recognize that other variables (e.g., human interference, interaction with other wild dog packs and densities of prey) are likely also to be important in governing wild dog habitat preference. As such, future studies should assess variables including kleptoparasitism and density of other predators and analyze these against wild dog habitat preference. Explicit investigation of the mechanisms linking availability of water and local habitat-wild dog associations will also be an important direction for future research. Nevertheless, our research advances current understanding of the linkages between wet and drought periods to wild dog home ranges, illustrating that water availability does not have a straight forward relationship with canids’ distribution. For example, dwindling water points may indirectly cause wild dogs to range wider, consequently encroaching into human settlements, which may lead to increased conflict with humans and exacerbated stress on this already endangered species.

## Supporting Information

S1 Table Water points. Water distribution points for years 1992 and 2000, coordinates are in WGS84 UTM ZONE 35S.
(XLS)

S2 Table Wild dogs. Wild dogs presence only data for years 1992 and 2000, coordinates are in WGS84 UTM ZONE 35S.
(XLS)
